# Heterogeneous catalytic ozonation of ciprofloxacin in aqueous solution using a manganese-modified silicate ore†

**DOI:** 10.1039/c8ra06880a

**Published:** 2018-10-01

**Authors:** Lisha Luo, Donglei Zou, Dongwei Lu, Bingjing Xin, Ming Zhou, Xuedong Zhai, Jun Ma

**Affiliations:** Key Laboratory of Ministry of Education for Groundwater Resources and Environment, College of New Energy and Environment, Jilin University Changchun 130000 P. R. China; Jilin Institute of Chemical Technology Jilin 130022 P. R. China; State Key Laboratory of Urban Water Resource and Environment, Harbin Institute of Technology Harbin 150090 P. R. China lvdongwei@hit.edu.cn

## Abstract

Manganese modified silicate ore (MnSO) prepared using an impregnation method was used as a heterogeneous ozonation catalyst, and the catalytic activity was evaluated by the degradation of ciprofloxacin (CIP). The results showed that the manganese oxide was successfully loaded onto natural silicate ore (SO). The degradation and mineralization efficiencies of CIP were considerably improved in the presence of MnSO. Under optimal conditions, the CIP removal process followed the pseudo-first-order reaction model well. The degradation rate constant of MnSO/O_3_ was 1.7 times and 3.3 times higher than those of SO/O_3_ and only O_3_, respectively. During the ozonation of the CIP aqueous solution in the presence of MnSO, the TOC removal rate reached 61.2% at 60 min, but was only 30.8% using ozonation alone. The addition of *tert*-butanol (TBA) significantly inhibited the degradation efficiency of CIP, which indicated that catalytic ozonation of MnSO followed a hydroxyl radical (·OH) reaction mechanism. Furthermore, MnSO showed great stability and durability over several reaction cycles.

## Abstract 2

Manganese-modified silicate ore showed remarkable catalytic oxidation activity for ciprofloxacin degradation and the corresponding mechanism was revealed.

## Graphical abstract

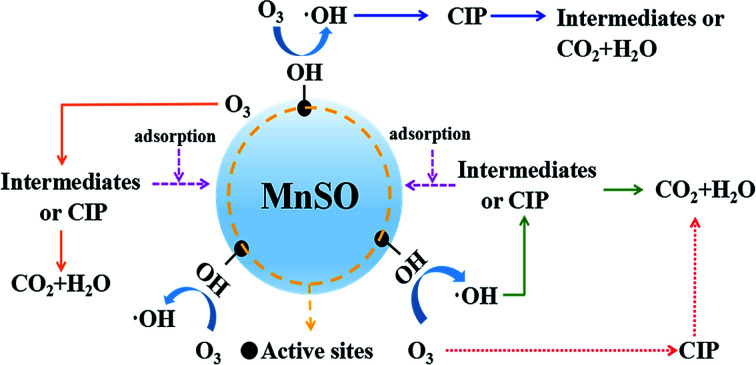

## 1. Introduction

In recent years, antibiotics have been widely developed and increasingly used in the aquaculture, animal farming, and human pharmaceutical industries.^1,2^ Most antibiotics enter aquatic systems and lead to chemical pollution and resistant gene pollution.^3^ Ciprofloxacin (CIP), as the second generation of fluoroquinolones, has been extensively used because of its capacity for efficient disease control and enhancing animal growth.^4,5^ The level of CIP concentration detected in the environment has increased from ng L^−1^ to mg L^−1^.^6^ Conventional biological wastewater treatment processes used in sewage treatment plants (STPs) are not effective for the removal of CIP because of its poor biodegradability.^7^ Therefore, it is urgent and necessary to develop an effective technology to remove CIP.

Heterogeneous catalytic ozonation, which combines ozone with a solid catalyst, is an effective advanced oxidation technology for the degradation and mineralization of CIP.^8–11^ The solid catalyst can accelerate the decomposition of ozone to hydroxyl radical (·OH) and can also adsorb organic pollutants on its surface to accelerate subsequent reactions between the ·OH and pollutant.^8^ Manganese oxide is among the most widely studied metal oxides as an ozonation catalyst because of its unique characteristics, such as environmental friendliness, diverse crystallographic structure, facile fabrication, and abundance in soil.^12–14^ As demonstrated in this study, manganese oxide supported by various porous materials (carbon-based multi-pore materials,^15,16^ honeycomb ceramics,^17^ and mesoporous molecular sieve^18^) can effectively enhance mechanical performance and provide more catalytic active sites. In addition, porous materials supported by manganese oxide can be easily separated, avoiding the waste of catalyst and secondary pollution. However, the carbon-based material is easily oxidized by ozone and can generate a large quantity of residual sludge that needs to be processed.^19–21^ In addition, the production of synthetic materials may cause certain pollution problems to the environment and increase costs.^22^ These disadvantages limit the application of carbon-based materials and synthetic catalysts in full-scale systems.

Natural mineral materials have been reported as heterogeneous ozone catalysts or supports because they are environmentally friendly and of low cost. Previous studies have indicated that natural mineral materials and their modified forms have been investigated for their potential to catalyze the ozonation of various organic contaminants in water and wastewater including bauxite,^21^ magnetite ore,^22^ brucite,^23^ goethite,^24^ and perovskite.^25^ However, the specific surface area of some natural mineral materials is small, and the adsorption performance of the target materials is poor;^21–25^ thus, they provide limited catalytic active sites. Therefore, it is necessary to seek a natural mineral material that simultaneously possesses a large surface area and a strong adsorption capacity for catalytic ozonation of pollutants.

In this study, we used a type of natural silicate ore as the support to fabricate a composite catalyst. MnO_*x*_ was selected as the active component of the catalyst. A composite catalyst was prepared using the impregnation method, and the MnSO obtained was characterized using different techniques. The influence of catalytic dose, solution pH, CIP concentration, and the reaction temperature on the degradation and mineralization of CIP were studied. Furthermore, a possible reaction mechanism for ozonation of CIP with MnSO is discussed.

## 2. Materials and methods

### 2.1 Chemicals

Silicate ore was obtained from advanced oxidation technology research center of the Harbin Institute of Technology. CIP was purchased from Sigma-Aldrich (≥98%). Manganese nitrate was purchased from the Tianjin Baishi Chemical Reagent Co., Ltd, *tert*-butyl alcohol (TBA) was purchased from the Tianjin Ruijinte Chemical Reagent Co., Ltd. Sodium thiosulphate was supplied by the Tianjin Reagent Chemical Co., Ltd. All chemicals used in this study were analytic grade and used without further purification. Deionized water was used during the experiment. The pH of the solution was adjusted with nitric acid and sodium hydroxide.

### 2.2 Preparation of MnSO catalysts

Modified natural silicate ore was prepared using an impregnation method. Detailed preparation information is as follows. First, the natural silicate ore particles were washed with deionized water several times to remove surface dust, and then grinded to 60–80 mesh through a high-speed universal mill. Second, 5 g of crushed natural silicate ore was soaked in Mn(NO_3_)_2_ (0.5 mol L^−1^, 100 mL) solution, and oscillated at a constant speed (120 rpm) for 24 h at 30 °C. Following precipitation and filtration, the silicate ore was dried at 70 °C for 36 h before calcination treatment at 500 °C for 5 h in air. Then, the MnSO samples were cooled to room temperature and stored in a desiccator before use.

### 2.3 Catalyst characterization

Scanning electron microscopy (SEM, Hitachi SU8000, Japan) with an acceleration voltage of 15 kV was used for analyzing the morphology of the samples, and the catalyst surface was sputter coated with gold. The crystalline shape of the catalyst was analyzed using X-ray diffraction (XRD, D/max-RA, Rigaku), operating with a Cu anode at 40 kV and 100 mA. The 2 theta data from 10° to 80° were obtained at a scanning speed of 0.03° s^−1^. The specific surface area, total pore volume, and average pore size of the catalysts were measured using a nitrogen gas adsorption analyzer (ASAP-2020, Micromeritics, USA) based on the Brunauer–Emmett–Teller (BET) method. Infrared spectra of the catalysts were measured under ambient conditions using a Fourier transform infrared (FT-IR) spectrum instrument (Spectrum 2000, Perkin Elmer, USA). X-ray photoelectron spectroscopy (XPS, Kratos-AXIS ULTRA DLD) using Al Kα as the source was used to determine the surface components of the composite. The characterization results of SEM, XRD, BET and FT-IR are provided in ESI (Fig. S1–S4 and Table S1 †).

### 2.4 Catalytic ozonation procedure

Batch catalytic ozonation degradation experiments of CIP were conducted in a 500 mL flat bottom beaker. A microporous titanium diffuser was connected at the bottom of the reactor to diffuse the ozone gas entering the reactor. Ozone was produced *via* a DHX-I ozone generator (Harbin Jiujiu Electrochemistry Technology Co., Ltd., China). The inlet ozone gas concentration was approximately 0.4 mg min^−1^ and the ozone flow rate was 300 mL min^−1^. During each semi-intermittent experiment, 400 mL of CIP aqueous solution (20 mg L^−1^) and a certain quality catalyst were added to the beaker. During the whole process, the slurry was continuously stirred to ensure that the catalyst was completely fluidized in the reaction solution. The experiment was conducted at 20 °C using a cryostat. The water samples were collected at regular intervals and filtered through cellulose acetate filters (0.22 μm) before analysis of CIP and total organic carbon (TOC) concentrations. A small amount of sodium thiosulfate solution was added as the termination agent. To test the stability of the MnSO, the catalyst was collected by centrifugal separation after the catalytic ozonation reaction, washed several times with deionized water, and dried at 70 °C for 24 h before reuse. The pH value of the solution was regulated using normal HNO_3_ and NaOH in all reactions for a comparison. To obtain effective experimental data, the experiment was repeated three times, and the results were averaged.

### 2.5 Analysis methods

CIP concentration was analyzed using high-performance liquid chromatography (HPLC; Agilent 1200 Series, DAD detector and XDB-C18 column) with a UV detector at 276 nm. The mobile phase was acetonitrile and 0.1% formic acid (88 : 12, v/v) with a 1.0 mL min^−1^ flow rate.

The first-order model was used to simulate the degradation kinetics of CIP, *C*_*t*_/*C*_0_ = exp(−*kt*). Here, *k* (min^−1^) is the pseudo-first-order rate constant of CIP; *t* (min) is the reaction time; and *C*_0_ and *C*_*t*_ denote the CIP concentrations in the solution at the initial concentration and a specific time point, respectively.

Total organic carbon (TOC) was measured using a total organic carbon analyzer. (TOC-VCPH, Shimadzu Japan). The concentration of leaching manganese was measured using inductively coupled plasma (ICP) (Perkin-Elmer optima 5300DV).

## 3. Results and discussion

### 3.1 Characterization of catalysts

The morphologies of the SO and MnSO were observed by scanning electron microscope (Fig. S1†). As shown in Fig. S1a,† the surface of the SO was rough and uneven. Many particles were piled atop one another and interrelated. Pores were abundant. As shown in Fig. S1b,† the surface of the MnSO gradually smoothed with the MnO_*x*_ introduction, probably owing to the MnO_*x*_ on the surface of the SO.

**Fig. 1:**
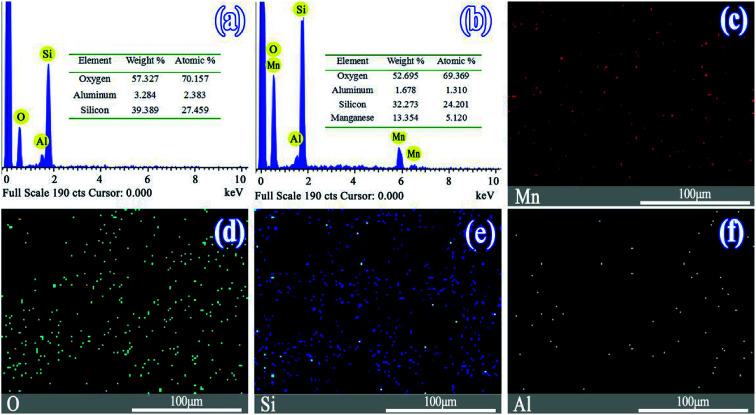
EDS spectrum of (a) SO, (b) MnSO, and (c)–(f) EDS mapping images of the MnSO.

**Fig. 2:**
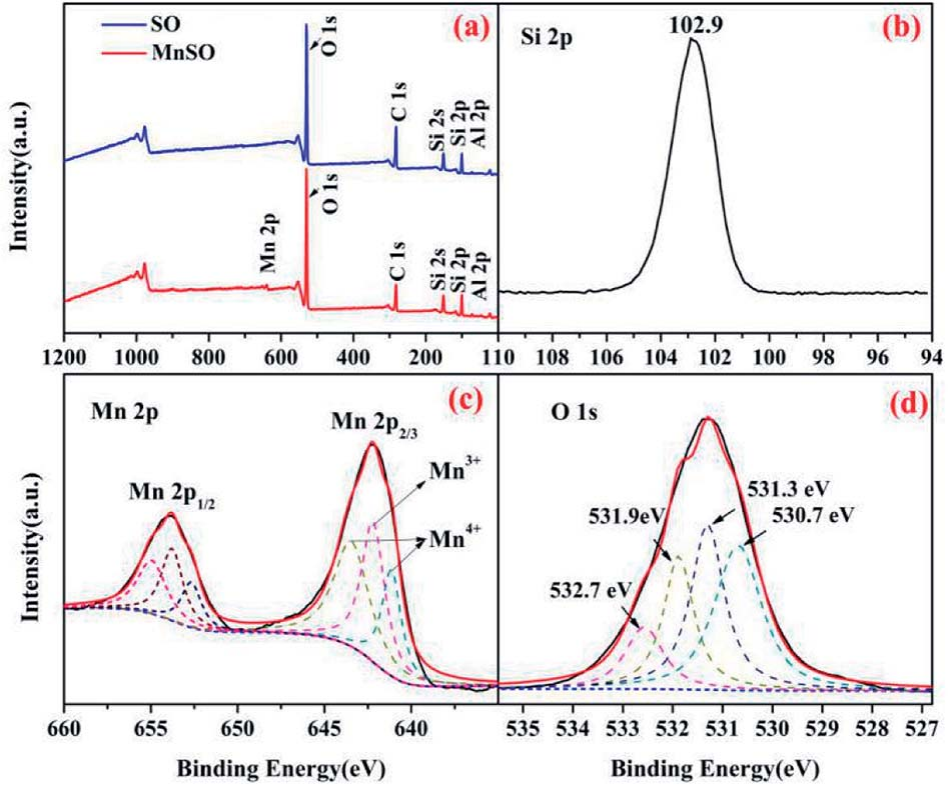
XPS spectra analysis of the SO and MnSO catalyst. (a) Wide-range scan of the SO and MnSO; (b) Si 2p, (c) Mn 2p, and (d) O 1s of the MnSO composites.

Fig. 1a and b show the EDS images of the SO and MnSO. The results confirmed the presence of the Mn element in the MnSO. The mass ratio of the surface Mn element for MnSO was 13.35%. Based on the EDS mapping image as shown in Fig. 2c the manganese was uniformly distributed on the MnSO surface. The formation of metal oxides and their effective distribution play an important role in MnSO degrading refractory organic pollutants.^26^ The results indicated that the Mn element was successfully doped in the SO catalyst. XRD of the SO and MnSO sample is shown in Fig. S2.† In the pattern of the SO, the diffraction peaks matched with the published XRD pattern of the cristobalite (JCPDS no. 89-3607) and α-quartz (JCPDS no. 85-1054). Compared to the SO, there were some unique diffraction peaks (2*θ* = 28.8°, 37.5°, 56.8°, 32.9° and 55.1°) which corresponded to the (110), (101), (221), (222), and (440) faces, respectively, on the MnSO sample. These diffraction peaks belong to MnO_2_ and Mn_2_O_3_.^27,28^ Therefore, it can be deduced that the MnO_*x*_ were effectively loaded on the SO surface.

The surface elemental composition and chemical states of the SO and MnSO were further investigated using XPS. As shown in Fig. 2a, compared to the characteristic peaks of the SO, there not only were characteristic peaks corresponding to Si 2p, C 1s, O 1s and Al 2p, but also to Mn 2p in the XPS spectrum of the MnSO composites, which confirms the presence of Mn in the MnSO composites. Fig. 2b shows the peak at 102.9 eV corresponding to Si 2p.^29^Fig. 2c shows the spectrum of Mn 2p_3/2_ shows three peaks, corresponding to MnO_2_ (641.1 and 643.4 eV) and Mn_2_O_3_ (642.2 eV),^30,31^ which were consistent with the aforementioned XRD results. This indicated that the MnO_2_ and Mn_2_O_3_ were successfully doped into the SO. As shown in Fig. 2d, the O 1s peaks at 530.7 eV, 531.3 eV, 532.7 eV, and 531.9 eV were assigned to the Mn–O, Al–O, Si–O, and OH(ads), respectively. The OH(ads) is beneficial for the generation of ·OH in catalytic ozonation.^32^

As shown in Fig. S3,† the N_2_ adsorption–desorption isotherms of the SO and MnSO were both identified as type IV according to the International Union of Pure and Applied Chemistry classification. This means that the SO and MnSO catalyst surface were mesoporous structures.^33^ The BET of both the SO and MnSO are listed in Table S1.† The specific surface areas of the SO and MnSO composite were determined to be 75.56 and 57.82 m^2^ g^−1^, respectively. The results showed that the loading of MnO_*x*_ resulted in a decrease in the average pore volume, specific surface area, and pore size of the SO. This illustrated that the manganese oxide dispersed into the pores of the SO and blocked them.

The FT-IR spectra were measured to investigate the surface function groups of the SO and MnSO. As shown in Fig. S4,† the absorption peak at 1105 cm^−1^ originated from Si–O.^21^ The bands at 478 cm^−1^ were attributed to the bending and stretching modes of AlO_6_.^34^ The presence of water molecules was supported by the appearance peaks of the bending vibration absorption of H–O–H at 1635 cm^−1^ and the stretching mode stretching vibration of O–H at 3419 cm^−1^.^35,36^

### 3.2 Adsorption and catalytic ozonation of CIP by MnSO

**Fig. 3:**
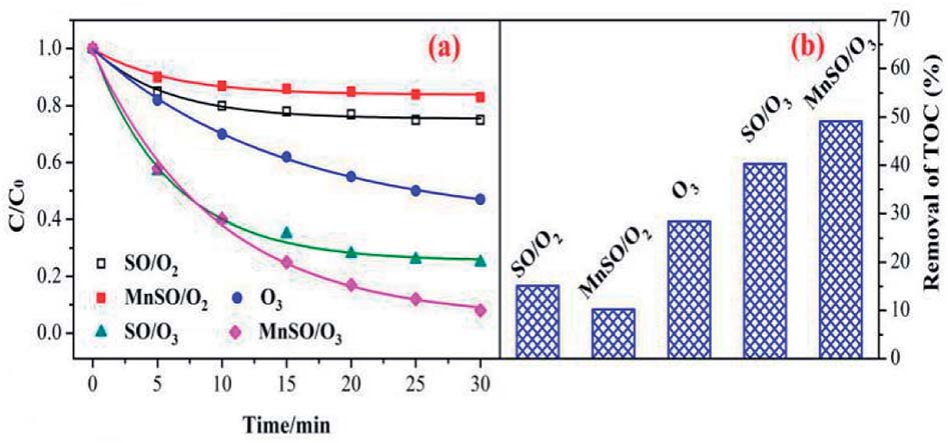
Degradation and mineralization of CIP by ozonation alone and with MnSO. Experimental conditions: pH = 7.0; *C*_0_ = 20 mg L^−1^; ozone gas flow rate = 0.3 L min^−1^; ozone gas concentration = 0.4 mg min^−1^; catalyst dose = 0.5 g L^−1^; *T* = 20 °C.

To evaluate the catalytic activity of the catalyst, the degradation and mineralization of CIP under different experimental conditions including adsorption, single ozonation, and catalytic ozonation experiments were determined as shown in Fig. 3. After 30 min, the adsorption efficiencies in the presence of SO and MnSO were approximately 25.3% and 17.5%, respectively. This type of natural SO and MnSO had good adsorption performance for the CIP, which benefited further ozonation reactions. The degradation of CIP reached 53.% over 30 min using a single ozonation process, whereas it increased to 74.9% and 92.1% by adding SO and MnSO during the catalytic ozonation process, respectively. The results showed that MnSO was an effective heterogeneous ozonation catalyst, which increased the degradation efficiency of CIP by approximately 39.1% as compared to the single ozonation process. As shown in Table S2,† the CIP degradation rates by adsorption, single ozonation, and catalytic ozonation followed the pseudo-first-order kinetic model. The CIP degradation rate constant with MnSO/O_3_ was 1.7 times higher than that of SO/O_3_, and 3.3 times higher than that of single ozonation. The results show that MnSO enhanced the catalytic activity in the ozonation of CIP. In comparison with the catalyst of MnO_*x*_/MNCNT^16^ and MnO_*x*_/MCM-41 (ref. 18) for refractory pollutant oxidative degradation, MnSO in this work was relatively high efficient, widespread, easily prepared and low cost, which has a better application prospect.

In addition, the TOC removal efficiency obtained over 60 min during the MnSO catalytic ozonation process (61.2%) was significantly higher than that during the SO catalytic ozonation (53.6%) and single ozonation (30.8%) processes. The adsorption process still had a great effect on the removal of TOC. The improvement of CIP degradation and TOC removal may have been a result of the following. First, the MnSO catalyst had a good adsorption capacity which could accelerate subsequent reactions between the ozone and pollutant.^37^ Second, the presence of the MnSO catalyst could promote decomposition of ozone to a hydroxyl radical (·OH).^8^ The ·OH was non-selective and could enhance the CIP and TOC removal. However, the removal rate of TOC was significantly lower than that of the degradation of CIP. This result was because a large number of intermediates produced during the CIP degradation process were difficult to degrade *via* ozone. Although CIP can be largely removed, the intermediate products cannot be completely converted into CO_2_ and H_2_O, thus reducing the removal effect of the TOC.

### 3.3 Influence of the catalyst dosage on the catalytic ozonation process

**Fig. 4:**
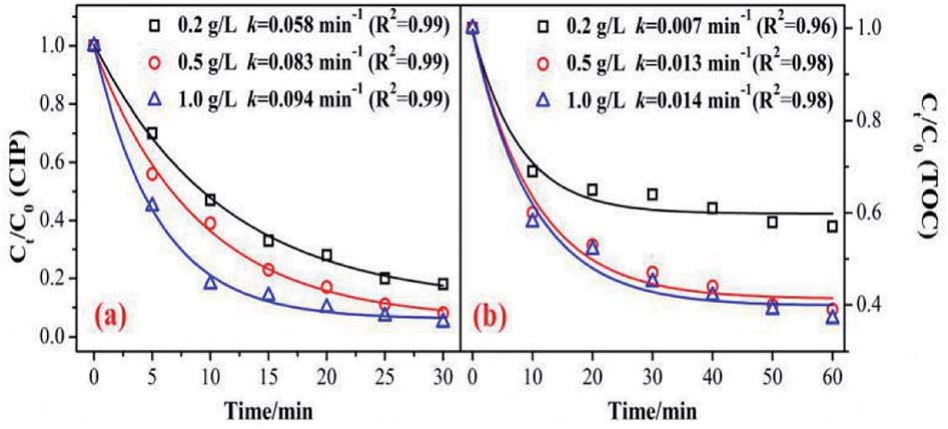
Effect of catalyst dosage on the removal of CIP and TOC. (Experimental conditions: pH = 7.0; *C*_0_ = 20 mg L^−1^; ozone gas flow rate = 0.3 L min^−1^; ozone gas concentration = 0.4 mg min^−1^; *T* = 20 °C).

The catalyst dosage relates to the reaction rate of the ozone with an organic reaction, and also relates to the water treatment cost in practical application. Therefore, it is necessary to discuss the optimum amount of catalyst. As shown in Fig. 4, the degradation and mineralization efficiency of CIP significantly increase with an increase in catalyst dose. The CIP removal efficiencies improved from 82% to 92.5% and the removal efficiency of the TOC increased by 20%.

The CIP degradation kinetic constants during the MnSO/O_3_ process were enhanced from 0.058 min^−1^ to 0.094 min^−1^ as the catalyst dosage increased from 0.2 g L^−1^ to 1.0 g L^−1^. Simultaneously, the TOC removal rate constants by MnSO/O_3_ also increased from 0.007 min^−1^ to 0.014 min^−1^ with the catalyst dosage increasing from 0.2 g L^−1^ to 1.0 g L^−1^. These results indicated that increasing the amount of catalyst in the solution was conducive to improving the specific surface area of the catalyst in the solution and facilitating decomposition of ozone molecules into more ·OH.^38^ However, the removal efficiency and mineralization efficiency of CIP were not significantly improved when the addition of the catalyst was 1.0 g L^−1^ because of the limited amount of ozone in the system. In addition, when the catalytic dose continued to increase, the particles collided with each other, thus affecting the effective contact between the catalyst and ozone.

### 3.4 Influence of CIP initial concentration on catalytic ozonation process

**Fig. 5:**
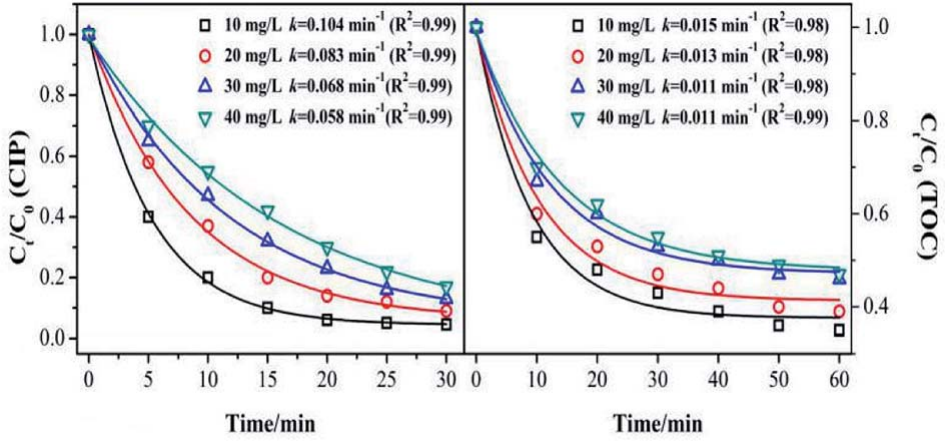
Effect of initial concentration of the target on removal of CIP and TOC. (Experimental conditions: pH = 7.0; ozone gas flow rate = 0.3 L min^−1^; ozone gas concentration = 0.4 mg min^−1^; catalyst dose = 0.5 g L^−1^; *T* = 20 °C).

Fig. 5 shows the effect of the initial concentration on the CIP and TOC removal during the MnSO catalytic ozonation process. It can be seen that the CIP or TOC removal decreased with increasing initial concentration. The CIP degradation kinetic constant was 0.104 min^−1^ with the initial concentration of 10 mg L^−1^, and it was approximately twice that with an initial concentration of 40 mg L^−1^. This result indicates that when the initial concentration of CIP was low, the amount of dissolved ozone in the solution and the amount of ·OH from the decomposition of ozone in the solution were high. They can rapidly degrade CIP and also result in a higher mineralization effect. When the initial concentration of CIP increases, the ozone is rapidly utilized by the high concentration of CIP. At the same time, the intermediate product competes with ozone for CIP, thus the degradation effect of CIP was significantly reduced.^39^

The removal rate of the TOC corresponded to the trend in the catalytic ozonation degradation rate of CIP, but it was still much lower than that of the CIP degradation. It can be inferred that the speculation produced a large number of intermediates that were more resistant to ozonation than CIP.

### 3.5 Influence of reaction temperature on the catalytic ozonation process

**Fig. 6:**
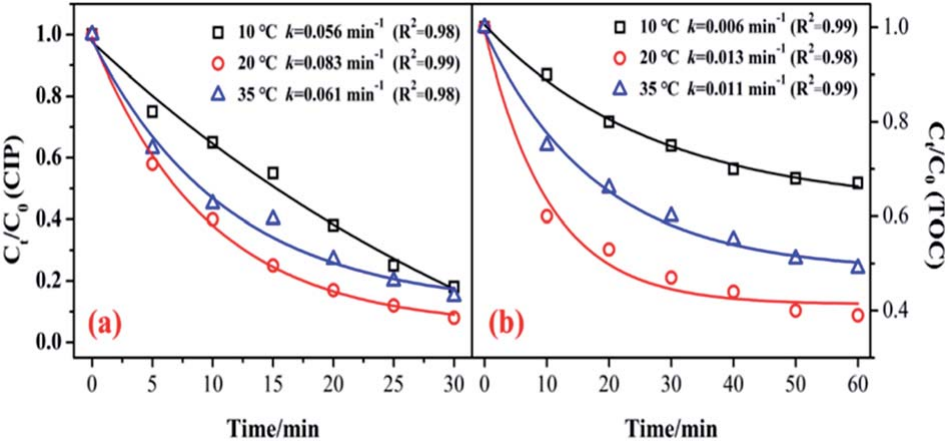
Effect of reaction temperature on the removal of CIP and TOC. (Experimental conditions: pH = 7.0; *C*_0_ = 20 mg L^−1^; ozone gas flow rate = 0.3 L min^−1^; ozone gas concentration = 0.4 mg min^−1^; catalyst dose = 0.5 g L^−1^).

Fig. 6 shows the influence of reaction temperature (10, 20, and 35 °C) on the catalytic activity of the MnSO for ozonation of CIP. As shown in Fig. 6, when the reaction temperature increased from 10 °C to 20 °C, the degradation efficiencies of CIP and TOC greatly increased from 82.3% to 92.1% and 33.1 to 61.3%, respectively. The kinetic information for CIP degradation and mineralization is shown in Fig. 6. It shows that when the temperature was 20 °C, the degradation rate was the fastest. However, when the temperature continued to increase to 35 °C, the removal efficiency was suppressed. Generally, a higher reaction temperature would lead to a faster reaction rate for a chemical reaction and mass transfer. In addition, a temperature increase can accelerate the decomposition of ozone and produce more hydroxyl radicals, which is beneficial to improving the catalytic ozonation effect. Nonetheless, the solubility of the ozone in water could decrease at higher temperatures. During the process of catalytic ozonation, the increase in reaction temperature results in these two opposing effects.^40,41^ Therefore, the reaction temperature of 20 °C has a positive effect on CIP degradation and mineralization during the process of ozonation of MnSO.

### 3.6 Influence of initial pH on the catalytic ozonation process

**Fig. 7:**
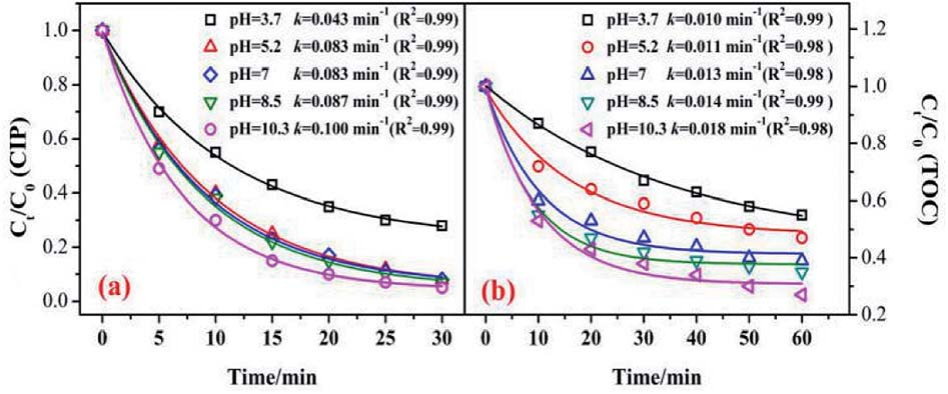
Effect of pH on the removal of CIP and TOC. (Experimental conditions: *C*_0_ = 20 mg L^−1^; ozone gas flow rate = 0.3 L min^−1^; ozone gas concentration = 0.4 mg min^−1^; catalyst dose = 0.5 g L^−1^; *T* = 20 °C).

In the catalytic ozonation system, the initial pH value was the key factor that affected the ozone decomposition rate and hydroxyl formation.^8,41^ In addition, the charge of the organic compounds in the solution varied under different pH conditions.^40,41^Fig. 7 shows the influence of the initial pH value on the CIP degradation and TOC removal efficiencies in catalytic ozonation. As shown in Fig. 7, the CIP removal efficiency clearly improved with an increase in the initial pH from 3.7 (79.5%) to 10.3 (95.8%). This was because in the alkaline solution more ·OH were yielded which was beneficial to organic decomposition.^8,42^ A similar trend was found in the TOC removal. At initial pH values of 3.7, 5.2, 7, 8.5, and 11.3, the TOC removals were 44.5%, 52.7%, 61.5%, 65.4%, and 73.2% after 60 min. The CIP degradation kinetic constants of the MnSO/O_3_ process were enhanced from 0.0043 min^−1^ to 0.100 min^−1^ as the pH increased from 3.7 to 10.3. Simultaneously, the TOC removal rate constants by MnSO/O_3_ also increased from 0.010 min^−1^ to 0.018 min^−1^ as the pH increased from 3.7 to 10.3.

Therefore, even though the intermediates of CIP were resistant to ozonation, they could be further oxidized or even mineralized by ·OH generated from the O_3_ decomposition. The increased production of ·OH during ozonation was an effective method to improve the TOC removal.

### 3.7 Catalyst stability and reusability

During the application process of the catalyst, the catalyst may have some problems such as the dissolution of the active component and damage to the active sites, which can lead to a significant decrease in its activity. Therefore, in the heterogeneous catalytic ozonation system, among the important measures to evaluate the effect of the catalyst was its reuse rate. Recycling experiments were conducted to evaluate the activity and stability of MnSO catalyst.

As shown in Table 1, the CIP and TOC removal efficiency slightly decreased with the increase in the catalyst reuse times. CIP degradation was reduced by *ca.* 3.0% and the TOC removal by *ca.* 2.9%. It was found that after four times of reuse, the MnSO catalyst still had good catalytic activity on CIP degradation and mineralization. The concentration of the released manganese ions in solution repeated over four times were detected to be 0.097 mg L^−1^, 0.088 mg L^−1^, 0.076 mg L^−1^, and 0.069 mg L^−1^, respectively. Although slight leaching of manganese ions was found, the catalytic capacity of the MnSO was hardly affected. The dissolution concentration of manganese ions was less than the limit in the drinking water of China (0.1 mg L^−1^).^43^ Thus, it had less effect on the safety of drinking water. We have characterized the catalyst by XRD after reactions (Fig. S5†). The results showed that the diffraction peaks of the catalyst remained unchanged before and after the reaction. This further demonstrated the good stability of the catalyst. Therefore, it could be concluded that the MnSO had great catalytic activity and stability, and is a promising catalyst for the removal of antibiotic wastewater.

**Table 1:** Stability and activity of the MnSO in the catalytic ozonation of CIP

Reuse time	CIP degradation^a^ (%)	TOC removal^b^ (%)	*c* (Mn)^a^/mg L^−1^
0	92.1	60.2	0.097
1	90.4	58.9	0.088
2	89.7	57.8	0.076
3	89.1	57.3	0.069

a Reaction time is 30 min.

b Reaction time is 60 min.

### 3.8 Possible reaction mechanism during the MnSO process

#### 3.8.1. Radical scavenging experiment

TBA is a well-known hydroxyl radical scavenger that can capture hydroxyl radicals produced during the process of catalytic ozonation decomposition. The reaction rate constant of TBA and hydroxyl radicals was 6.0 × 10^8^ M^−1^ S^−1^, which was much higher than the reaction rate constant of 3.0 × 10^−3^ M^−1^ S^−1^ for TBA and ozone.^12^ As shown in Fig. S6,† the effects of TBA on the degradation of CIP were investigated. After the addition of TBA (100 mg L^−1^), the CIP degradation was significantly inhibited. When 100 mg L^−1^ of TBA was added into the aqueous solution, reductions of 54.5% and 28.9% for the degradation efficiency during the processes of catalytic ozonation and ozonation alone at 30 min were noted, respectively. It was shown that during the process of degradation of CIP, TBA and CIP competition for ·OH produced intermediates of high selectivity and inertia, ending the chain reaction of free radicals.^41^ This hindered the reaction of ·OH and CIP, resulting in a reduction in the CIP degradation rate in the system. At the same time, it was also indirectly proven that the catalytic ozonation degradation of the CIP system by MnSO was the mechanism of hydroxyl radical action.

To further explore the reaction mechanism, O_3_ was replaced by the N_2_ in same experimental setup. As shown in Fig. S7,† under the MnSO/N_2_ system, the removal of CIP was 18.2%. When 100 mg L^−1^ TBA was added to the MnSO/N_2_ system, the removal of CIP remained unchanged. It indicated that there was no ·OH in the water, and the removal of CIP was probably due to the adsorption of catalyst. In comparison, MnCO/O_3_ system had significantly higher CIP removal. However, the addition of TBA greatly inhibited the degradation of CIP in MnCO/O_3_ system. Base on above experiment results, it can be concluded that O_3_ decomposition was the sole source of ·OH under MnSO catalytic ozonization.

#### 3.8.2 Mechanisms of catalytic ozonation by MnSO

**Fig. 8:**
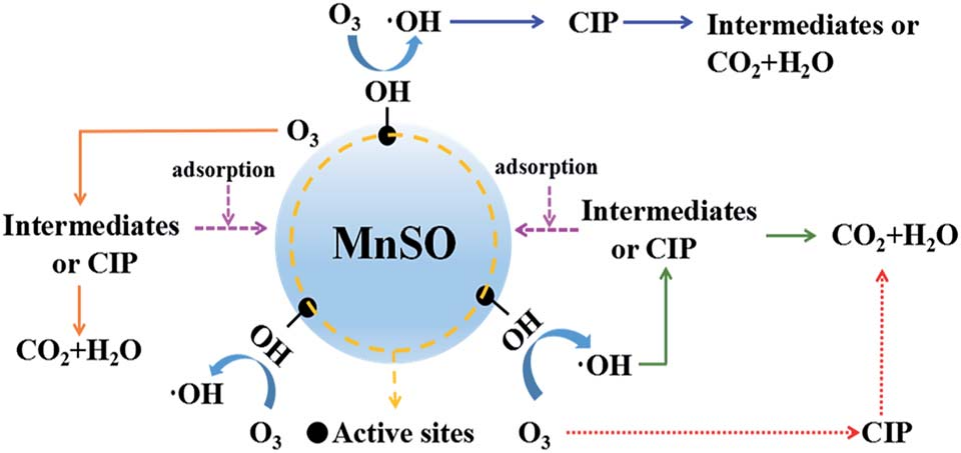
Reaction mechanism of the MnSO process.

From the aforementioned discussion, it was quite obvious that MnSO showed superior adsorption performance and perfect catalytic activity in heterogeneous catalytic ozonation to remove CIP. The oxidation reaction on the surface of the catalyst was the most important step in catalytic ozonation. When ozone or organic molecules were adsorbed on the catalyst surface, the oxidation reaction was significantly accelerated. To illustrate the possible interaction relation of ozone and the MnSO catalyst in the ozonation of CIP and its intermediates, a simplified mechanism showing the catalytic ozonation of CIP with the MnSO catalyst is shown in Fig. 8. Ozone can directly attack CIP in aqueous solution to generate CO_2_ and H_2_O, but the rate of direct reaction is much less than that of the oxidation of the hydroxyl radicals. The existence of ·OH resulted in successive oxidation reactions of CIP and its intermediates and promoted TOC removal.

The chain reactions of ozone decomposition were as followed:^19^

1 O_3_ + H_2_O → 2·OH + O_2_, *k*_2_ = 1.1 × 10^−4^ M^−1^ s^−1^

2 O_3_ + OH^−^ → O_2_˙^−^ + HO_2_˙, *k*_2_ = 70 M^−1^ s^−1^

3 O_3_ + ·OH → O_2_ + HO_2_˙ ↔ O_2_˙^−^ + H^+^

4 O_3_ + HO_2_˙ → 2O_2_ + ·OH, *k*_2_ = 1.6 × 10^9^ M^−1^ s^−1^

5 2HO_2_˙ → O_2_ + H_2_O_2_

Furthermore, the adsorption of CIP and the intermediates on the MnSO catalyst contributed to the increase in the CIP and TOC removal, which is also beneficial to the subsequent oxidation process.

Consequently, in this study, there were three pathways for heterogeneous catalytic ozonation as follows:^41^ (1) chemisorption of ozone on the catalyst surface was decomposed by the formation of surface oxygenated radical species, which promoted the formation of hydroxyl radicals. The hydroxyl radicals rapidly removed CIP and the intermediates. (2) Organic molecules (CIP or intermediates) adsorbed on the surface of the catalyst were oxidized by gaseous or aqueous ozone. (3) Adsorption of both ozone and organic molecules (CIP or intermediates) on the surface of the MnSO catalyst and their direct and indirect reactions occurred between ozone and CIP, leading to a rapid degradation of CIP.

The reactions of CIP degradation can be simplified as:^44^

(I). Indirect (radical) reactions.

(a). Catalyst surface reaction:

6 MnSO^−·OH^ + CIP → MnSO + CO_2_+H_2_O + intermediates

7 MnSO^−·CIP^ + ·OH → MnSO + CO_2_+H_2_O + intermediates

(b). Bulk solution reaction:

8 CIP + ·OH → intermediates + CO_2_ + H_2_O

(II). Direct (molecular O_3_) reactions.

(a). Catalyst surface reaction:

9 MnSO^−·O_3_^ + CIP → MnSO + CO_2_+H_2_O + intermediates

10 MnSO^−·CIP^ + O_3_ → MnSO + CO_2_+H_2_O + intermediates

(b). Bulk solution reaction:

11 CIP + O_3_ → intermediates + CO_2_+H_2_O

In addition, according to the catalyst characterization results shown in Fig. 2 and S2,† different states of manganese on the surface of MnSO led to more electron transfer opportunities and promoted its high catalytic activity.^45^

## 4. Conclusions

Manganese modified natural SO catalysts were successfully prepared using the impregnation method and showed remarkable catalytic activity for the degradation and mineralization of CIP. The characterizations of the MnSO catalyst showed that MnO_*x*_ was distributed on the surface of the SO. The CIP removal rate with an MnSO catalyst at 30 min could reach 92.1%, but just 53% using O_3_ alone. The removal rate of TOC was 30.4% higher during the catalytic ozonation than with O_3_ alone. During MnSO catalytic ozonation, with the increase in catalyst dosage and the initial solution pH, the CIP and TOC removal efficiency in aqueous solution both increased. However, the capability of the catalyst for CIP degradation and TOC removal decreased with an increase in the initial concentration of CIP. The MnSO catalytic ozonation followed the ·OH production mechanism. The CIP removal was attributed to a synergistic effect of the adsorption, ozone oxidation, and ·OH oxidation. The activity of MnSO on CIP degradation and mineralization slightly decreased with repeated use several times. This was because of the leaching of the manganese ion from MnSO into solution, while the MnSO catalyst maintained excellent activity. This study may provide information on an inexpensive, viable, and promising catalyst towards effective catalytic ozonation of antibiotics from wastewater for practical application.

## Conflicts of interest

There are no conflicts to declare.

## Supplementary Material

RA-008-C8RA06880A-s001
