# Supplementary material for: Heterogeneous catalytic ozonation of ciprofloxacin in aqueous solution using a manganese-modified silicate ore
Source: RSC Adv. 2018 Oct 1;8(58):33534–41. doi: 10.1039/c8ra06880a (PMC9086485; doi:10.1039/c8ra06880a)
Supplement: RA-008-C8RA06880A-s001 [file RA-008-C8RA06880A-s001.pdf]

Electronic Supplementary Material (ESI) for RSC Advances.
This journal is © The Royal Society of Chemistry 2018

Supporting Information

Heterogeneous catalytic ozonation of ciprofloxacin in aqueous solution using a manganese-modified silicate ore

Lisha Luo[ab], Donglei Zou[a], Dongwei Lu[c]*, Bingjing Xin[b], Ming Zhou[b], Xuedong Zhai[c] and Jun Ma[c*]

[a] *Key Laboratory of Ministry of Education for Groundwater Resources and Environment, College of New Energy and Environment, Jilin University, changchun 130000, P. R. China*

[b] *Jilin Institute of Chemical Technology, Jilin130022, P. R. China*

[c] *State Key Laboratory of Urban Water Resource and Environment, Harbin Institute of Technology, Harbin 150090, PR China. E-mail: lvdongwei@hit.edu.cn*

Table S1 BET of the SO and MnSO

Sample	BET (m^2 g^{-1})	Pore volume (cm^3 g^{-1})	Average pore size (nm)
SO	75.56	0.24	12.95
MnSO	59.11	0.16	10.9

Table S2 First-order kinetic parameters of the CIP removal during different oxidation processes

	k (min^{-1})	R^2
SO/O_2	0.014	0.9934
$MnSO/O_2$	0.009	0.9940
O_3	0.025	0.9993
SO/O_3	0.043	0.9955
$MnSO/O_3$	0.083	0.9968

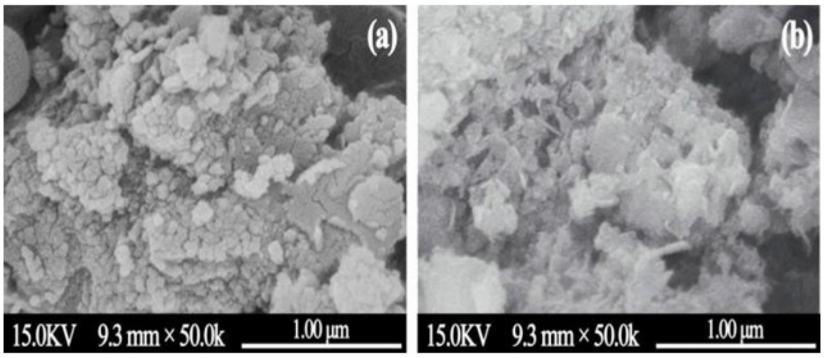

Fig. S1 SEM images of the samples SO (a) and MnSO (b).

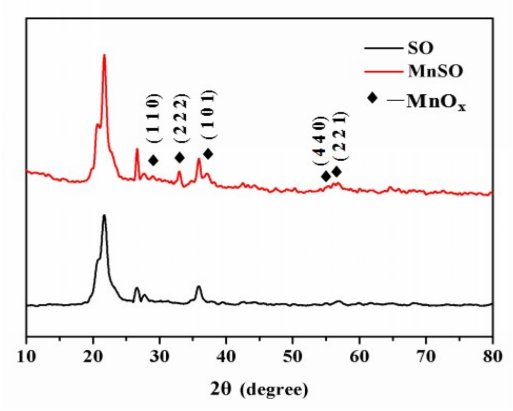

Fig. S2 XRD of the SO and MnSO catalyst.

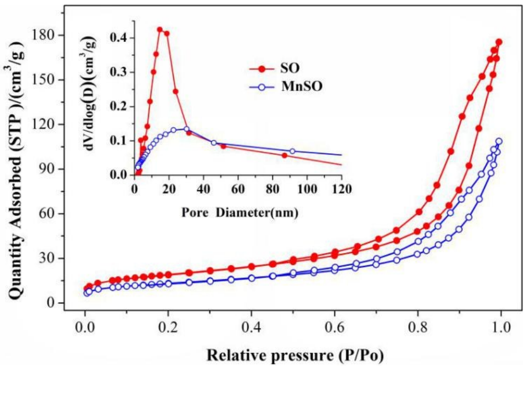

Fig. S3 N_2 adsorption-desorption isotherms of the SO and MnSO

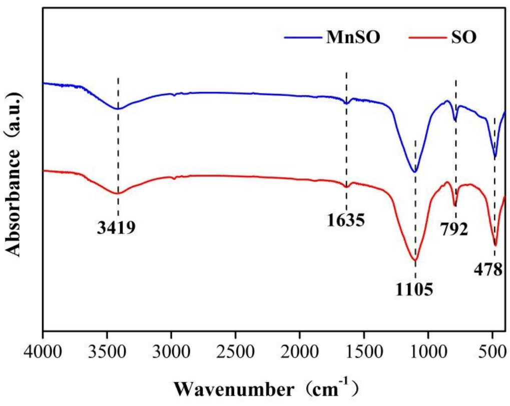

Fig. S4 FTIR spectra of SO and MnSO.

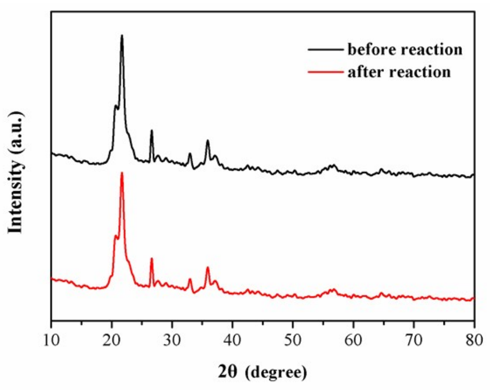

Fig. S5 XRD of MnSO before and after catalytic ozonization of CIP

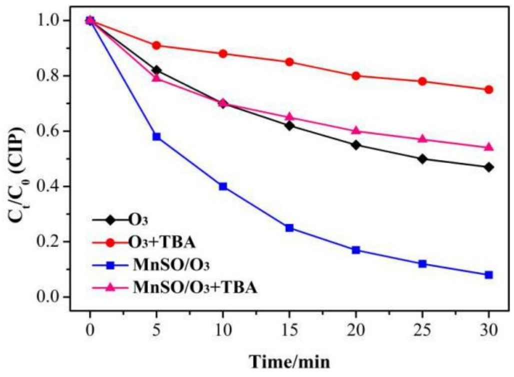

Fig. S6 Effect of TBA on CIP degradation.

(experimental conditions: pH = 7.0, C_0 = 20 mg L^{-1}; ozone gas flow rate = 0.3 L min^{-1}; ozone gas concentration = 0.4 mg min^{-1}; catalyst dose = 0.5 g L^{-1}; T = 20 °C; TBA=100 mg L^{-1}).

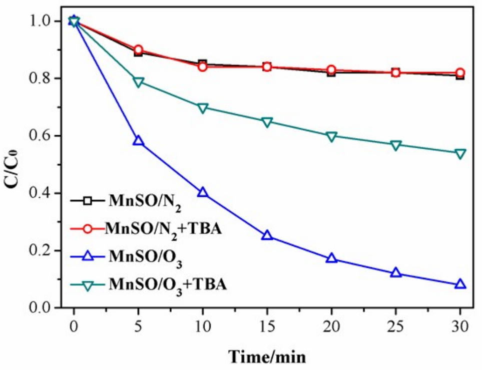

Fig. S7 The system of replace O_3 by N_2 on CIP degradation.

(experimental conditions: pH = 7.0, C_0 = 20 mg L^{-1}; N_2 flow rate = 0.3 L min^{-1}; catalyst dose = 0.5 g L^{-1}; T = 20 °C; ozone gas flow rate = 0.3 L min^{-1}; ozone gas concentration = 0.4 mg min^{-1}; TBA=100 mg L^{-1}).
